# Running on Empty: Self-Reported Sleep/Wake Behaviour during Ultra-Marathon Events Exceeding 100 Miles

**DOI:** 10.3390/ejihpe12070058

**Published:** 2022-07-11

**Authors:** Dean J. Miller, Darren Bianchi, Michele Lastella

**Affiliations:** Appleton Institute for Behavioural Science, Central Queensland University, Adelaide, SA 5034, Australia; darren.bianchi@cqumail.com (D.B.); m.lastella@cqu.edu.au (M.L.)

**Keywords:** ultra-marathon, sleep, sleep deprivation, athletes

## Abstract

The aim of this study was to examine sleep/wake behaviour and sleep strategies before, during and after ultra-marathon running events exceeding 100 miles (161 km). A total of 119 athletes completed a web-based questionnaire regarding their habitual sleep/wake behaviour before, during, and after ultra-marathon participation. Event-specific data were grouped by race distance categories; 100–149 miles (161–240 km), 150–199 miles (241–321 km), and ≥200 miles (322 km). Athletes commonly reported not sleeping throughout the duration of their races (74%). However, for events that were ≥200 miles, athletes reported more sleep opportunities, longer sleep duration, and more total sleep when compared to events that were 100–149 miles in distance (*p* ≤ 0.001). This suggests that for races of shorter distances, the benefit of continuous racing outweighs the negative impact of continuous wakefulness/sleep deprivation. However, for longer races (≥200 miles), there is an apparent tradeoff between sleep deprivation and race strategy, whereby athletes cannot sustain a desired level of performance without obtaining sleep. This is consistent with established sleep/wake behaviour models suggesting that sleep need increases as wakefulness increases, or in this case, as race duration increases. For athletes participating in ultra-marathons, sleep management education and/or consultation with a sleep scientist prior to racing may be beneficial. Future research should examine the optimal strategies concerning the frequency and duration of sleep during ultra-marathons and the subsequent impact on performance.

## 1. Introduction

Ultra-marathons are foot races usually completed on off-road courses longer than the traditional marathon distance [1,2]. Due to the extreme distances covered in these races, their duration often impedes on the human body’s 24 h sleep/wake rhythm [3,4]. That is, athletes are competing during the hours of the day in which they would usually be sleeping [5]. Understanding sleep management practices is important for athletes during all phases of ultra-marathon competition (i.e., pre-race, in-race and post-race) [6,7,8,9]. Sleep deprivation is a major concern for athletes competing in ultra-marathon races, particularly in events beyond 100 miles (160 km) [6]. Appropriate sleep strategies prior to anticipated sleep deprivation have been suggested to provide some protection against the degrading effects of forgoing sleep [10]. A wide array of sleep strategies are available to the general public [11], however, these need to be contextualised into the extreme environment of ultra-marathon competition.

Sleep extension (i.e., acquiring more sleep than habitually obtained) appears to be a popular sleep strategy for athletes in the preparation phase of ultra-marathon competition [6,7]. There is some evidence suggesting that sleep extension has a positive impact on sport-specific performance, as well as being a potentially effective tool in preparing for anticipated sleep deprivation [10,12]. Within the context of ultra-marathon competition, sleep extension appears to be the most appropriate sleep strategy for improving race performance [13]. Following competition, athletes may report sleep disturbances or reduced sleep quality [14]. Often, these disruptions are related to acute physiological or psychological changes associated with competition [14,15]. The importance of sleep in the recovery from athletic competition is well established [7,16,17]. As with the preparation phase of competition, sleep extension through increased nightly sleep duration and daytime naps is appropriate following competition [7,18,19].

Given the extreme nature of ultra-marathons, data related to the in-race sleep behaviour of ultra-marathon athletes is limited, particularly for events beyond 100 miles [6,13,20]. Currently, there is only one study that has acquired objective sleep data prior to, during, and after a race over 100 miles [1]. For this 200-mile race, athletes obtained 6.0 h of sleep in the week prior to the event, obtained a total of 4.7 h of sleep during the race (average race duration: 82.5 h), and obtained 6.3 h of sleep in the week following the event [1]. On average, athletes obtained their 4.7 h of sleep over 4.8 individual sleep opportunities. This is consistent with previous subjective findings indicating that when athletes reported having an in-race strategy for sleep, a preference for short sleep opportunities (i.e., naps) was common [6,13]. During a shorter ultra-marathon event of ~36 h duration, athletes were taking 1–2 naps, averaging 9 min of sleep (4–22 min range) [13]. It appears that athletes participating in events lasting >36 h often neglect or forgo sleep entirely to maximise “moving time” [6,20]. In contrast, 95% of athletes competing in events lasting longer than 60 h report sleeping on at least one occasion [6].

Currently, there are limited sleep data for athletes participating in events exceeding 100 miles [6]. To provide best practice sleep guidance to athletes in these extended duration events, an understanding of the sleep/wake behaviour and sleep strategies currently employed should be achieved. Importantly, this guidance is reliant on data from the three phases of competition (i.e., pre-race, in-race, and post-race). Therefore, the aim of this study was to examine the sleep/wake behaviour and sleep strategies of athletes before, during, and after ≥100 mile ultra-marathons.

## 2. Methods

### 2.1. Design

A self-report questionnaire was implemented to obtain data related to the sleep/wake behaviour and sleep strategies of athletes that have competed in ultra-marathon events ≥100 miles. The questionnaire was completed on a web-based platform (Qualtrics, Provo, Utah, USA) and was modelled upon the one used by Martin et al. [6]. Social media posts to ultra-marathon focused Facebook pages provided a brief overview of the aims and expectations of the study, accompanied by links to the questionnaire. Participants were requested to report demographic information, habitual sleep/wake behaviour, sleep behaviour, race details for ultra-marathon events (limited to longest three events ≥100 miles), experiences of sleep deprivation during events, and their sleep strategies before, during, and after ultra-marathon participation.

### 2.2. Participants

A total of 123 participants completed the web-based questionnaire. Data obtained from four of the participants were excluded due to incomplete or irrelevant responses (e.g., reporting the duration of the event in place of event distance where event distance was requested). Therefore, 119 responses were analysed in the final sample (29 females; age: 45.2 ± 9.8 years; age range: 26–68 years). All participants received a clear explanation of the study and provided informed consent electronically prior to participation. No incentive was offered for participating in this study. The study was approved in May 2021 by the Central Queensland University Human Research Ethics Committee (Ethics Code: 2021-018).

### 2.3. Measures

The following sleep variables were collected for habitual sleep behaviour:

- Bedtime (hh:mm): Self-reported clock time at which a participant went to bed to attempt to sleep;
- Sleep latency (min): Self-reported time between bedtime and sleep onset time;
- Total sleep time (hours): The amount of time spent in bed asleep during a night-time sleep period;
- Get-up time (hh:mm): Self-reported clock time at which a participant got out of bed.

Ultra-marathon-specific sleep behaviour questions required participants to record details for up to three ultra-marathon events exceeding 100 miles. For each event, athletes reported the race distance, the number of sleep opportunities, and the duration of each sleep opportunity (min). In addition, independently of the events reported, athletes indicated the locations where they had slept during ultra-marathon races (i.e., aid station, on-trail) and any indicators of sleep deprivation they may have experienced competing in ultra-marathons that were ≥100 miles. The questionnaire and indicators of sleep deprivation utilised were adapted from Hurdiel et al. [13] and allowed athletes to select multiple responses. Indicators included visual hallucinations, trips/falls due to inattention and difficulty keeping eyes open while running, difficulty keeping eyes open at any time, and difficulty recalling previous sections of the race. Finally, athletes were asked to briefly describe their strategy for sleep in the week before an event, during a race, and the week following an ultra-marathon event (pre-race, in-race, and post-race phases of competition). These open-ended responses were categorised based upon previously reported strategies [6]. Where appropriate, athletes’ responses were scored in more than one category.

### 2.4. Statistical Analysis

Data were screened before analysis, units of measurement for habitual sleep behaviour and in-race sleep behaviour were checked for accuracy and converted to the requested unit of measure where necessary. Open-end responses for athletes’ sleep strategies before, during, and after competition were scored in categories modelled on strategies previously reported [6].

A descriptive analysis of event-specific sleep behaviour and habitual sleep behaviour was also conducted. Event-specific sleep behaviour data were grouped by event distance; data were grouped as 100–149 miles (161–240 km), 150–199 miles (241–321 km), and ≥200 miles (≥322 km). Race-specific data were grouped in this manner in an effort to maximise the relevance to ultra-marathon athletes competing in the sport. The descriptive analysis of event-specific sleep data and habitual sleep behaviour from the present study is reported as mean ± standard deviation.

Kruskal–Wallis H tests were conducted to analyse the significance of the reported differences in event-specific sleep behaviour variables across the three event distance categories. Visual inspection of P-P plots indicated non-normal distributions for each event-specific sleep variable. The Kruskal–Wallis H test was selected due to it being a robust test for non-normal distributions and unequal sample sizes among categories [21]. Data were analysed using SPSS (version 27; IBM Corp, Armonk, NY, USA).

## 3. Results

### 3.1. Participants

Participants predominantly resided in the United States of America (45.4%) and Australia (44.5%). The remaining 10.1% of athletes resided in Canada, the United Kingdom, Great Britain and Northern Ireland, New Zealand, Sweden, and the Czech Republic. Ultra-marathon experience in ≥100 mile events ranged between one and thirty-nine events (5.9 ± 6.2 events). Habitual sleep behaviour indicated an average bedtime of 22:00 h ± 57 min and a waketime of 05:54 h ± 62 min, taking 15.5 ± 11.2 min to fall asleep and obtaining 7.0 ± 0.9 h of sleep per night. The 119 participants reported a total of 287 separate ultra-marathon events, with 188 events between 100–149 miles, 28 events between 150–199 miles, and 71 of ≥200 miles.

### 3.2. Sleep Strategies before, during and after Competition

**Figure 1:**
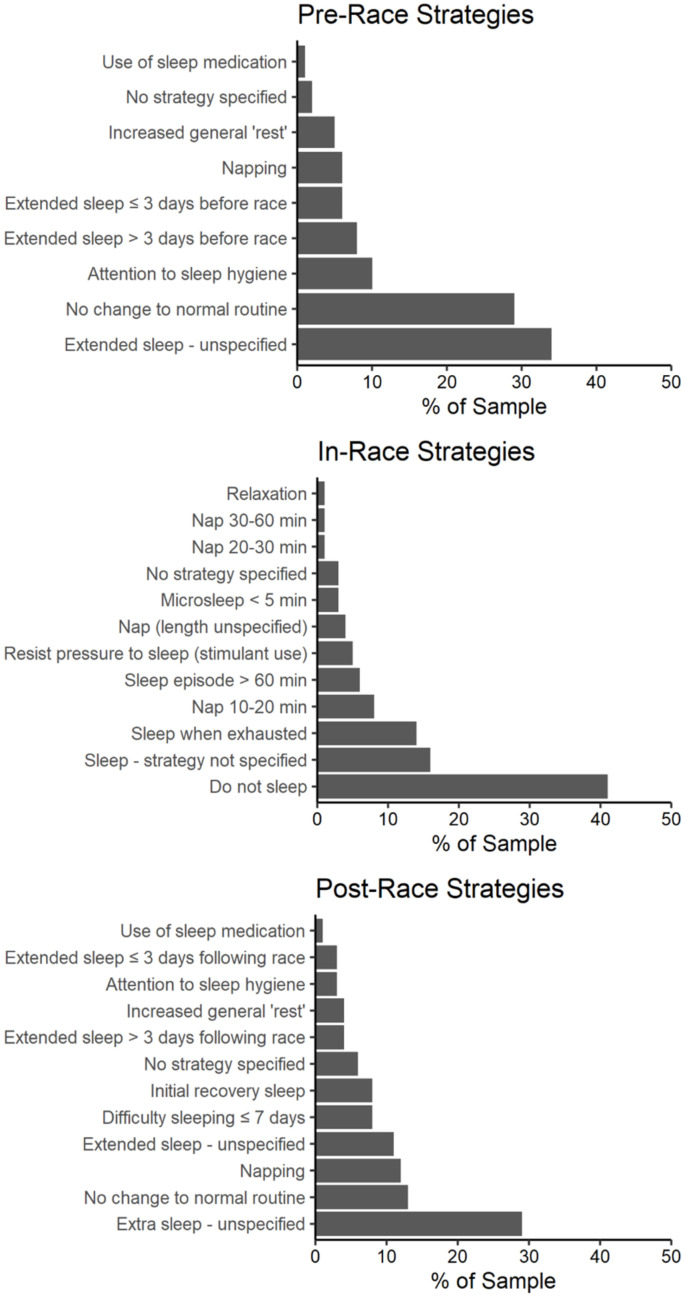
Sleep strategies before, during, and after ultra-marathon competition. Note: Sleep strategy categories are modified from Martin et al. [6]. Multiple strategies may have been indicated in athletes’ responses and were scored in multiple categories.

Descriptions of sleep strategies before, during, and after an event were reported independently of race distance categories. Participants may have indicated multiple strategies in their responses and, where appropriate, were scored in multiple categories. In the pre-race phase of the competition, participants indicated a preference for a sleep extension as their chosen strategy. Sleep extension was indicated in 48% of pre-race sleep strategies (Figure 1). In comparison, 29% of strategies involved no change to normal routine during the pre-race phase of competition (Figure 1). The most prevalent in-race sleep strategy was to avoid sleep altogether. Forty-one percent of the reported sleep strategies were to not sleep at all during the event; with a further 14% indicating that they would resist sleep until exhausted (Figure 1). Sleep or naps of an unspecified length were indicated in 20% of strategies. Where sleep length was included in a strategy, 10–20 min naps were most common (8%; Figure 1).

Following competition, the most common strategy was to obtain extra sleep, but in an unspecified fashion (29%; Figure 1). Where a sleep strategy was specified, the extension of night-time sleep opportunities (18%) and napping (12%) were most frequently employed (Figure 1). Interestingly, athletes also reported no change to the normal routine (13%) and a quick return to a normal routine after recovery sleep (8%; Figure 1).

### 3.3. In-Race Sleep/Wake Behaviour

For events that were ≥200 miles the average race distance was 219.8 ± 27.6 miles (353.7 ± 44.4 km), which took an average of 92.1 ± 23.8 h to complete. Athletes reported an average of 248.6 ± 228.0 min (i.e., 4.14 ± 3.8 h) of total sleep per event from 4.1 ± 3.2 individual sleep opportunities. On average, sleep opportunity length for ≥200 mile events was 59.3 ± 62.4 min, with sleep opportunities ranging between 0 and 360 min in duration. Only one athlete indicated obtaining no sleep during a ≥200 mile event.

Athletes competing in 100–149 mile events reported 12.1 ± 50.5 min of total event sleep, 0.5 ± 2.1 individual sleep opportunities, and an average sleep opportunity length of 10.6 ± 31.8 min. For this category, the average race distance completed was 105.6 ± 10.4 miles (170 ± 16.8 km), with an average of 30.9 ± 8.3 h race duration. Of the 188 races between 100–149 miles, no sleep was reported in 139 cases (74%). In-race sleep data for the three categories of race distance are presented in Table 1.

### 3.4. Sleep Locations and Indicators of Sleep Deprivation

Locations of sleep during events and athletes’ experiences of sleep deprivation indicators were reported independently of race distance categories. Athletes may have reported multiple sleep locations or experience of sleep deprivation indicators. Not all athletes reported sleep during events; therefore, a total of 110 responses for sleep location were made. The most reported sleep location was race-established aid stations with some form of bedding (45%; e.g., bed or stretcher). Other sleep locations included “dirt naps” on trails (25%), in vehicles (25%), and in a chair (5%).

Experiences of sleep deprivation were reported for 91% of all events. Visual hallucinations (26%) were the most common experience of sleep deprivation for athletes in the present study. Similar reporting rates were seen for difficulty keeping eyes open while running (19%) and difficulty staying awake at any time (20%) as experiences of sleep deprivation. Less common were difficulty recalling previous sections of the race (14%) and trips and falls due to inattention (12%). The final 9% of responses reported no indicators of sleep deprivation during their ultra-marathon events.

## 4. Discussion

The main finding of this study was that ultra-marathon competitors report severe sleep restriction during ultra-marathons exceeding 100 miles. Sleep deprivation was reported for 91% of the 287 events, with 74% of responses for events between 100–149 miles reporting no sleep at all. Resisting sleep is entirely consistent with previous findings showing that few athletes obtain any sleep during 100-mile events, or events of ≤36-h duration [6,20]. For athletes that sleep during a ~100-mile event, sleep is generally obtained during brief naps [6]. Again, this was supported by the outcomes of the present study, with athletes in the 100–149 mile event category reporting up to two individual sleep opportunities with an average duration of 10 min per sleep opportunity.

Consistent with previous findings [6], the athletes in the present study reported sleep extension as their preferred pre-race sleep strategy. Sleep extension has been shown to be effective in improving performance in ultra-marathon competition compared to other strategies such as training in a sleep-deprived state [13]. However, 29% of athletes in the present study indicated no change from their habitual sleep routine in the week prior to competition. This is concerning given that the habitual sleep of participants in this study was below the recommended ~8 h of nightly sleep [11]. Therefore, athletes that (1) do not habitually obtain >8 h sleep and (2) do not utilise sleep extension as a pre-race strategy are beginning period of sleep deprivation (i.e., ultra-marathon competition) in an already compromised state. It would be beneficial for athletes to maintain the recommended amount of habitual sleep as well as implementing sleep extension leading up to ultra-marathon competition.

**Figure 2:**
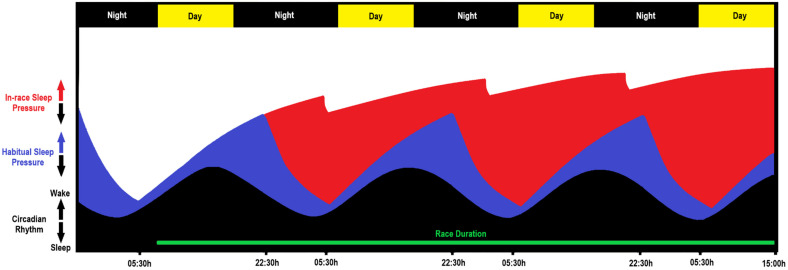
Schematic representation of an athlete completing a 200-mile ultra-marathon. Black fill represents circadian rhythm; blue fill represents habitual sleep; red fill represents in-race sleep; green bar represents race duration. The distance between the peak of black fill and the peak of the blue fill represents habitual sleep pressure experienced by the athlete, the distance between the peak of the black fill and the peak of the red fill represents the sleep pressure experienced during the race. This schematic does not account for potential shifts in circadian phase. Note: habitual and in-race sleep data used for this illustration were gleaned from Bianchi et al. [1], representations of circadian rhythm and sleep pressure are theoretical and not based on objective data from participants.

For in-race sleep, events ≥200 miles had an average of ~4 h of sleep and an average race duration of 92 h. Significantly less sleep was reported for shorter events, with events of ≥200 miles showing more sleep opportunities (~4 episodes) and longer average sleep opportunity duration (~59 min). Similar time-related increases in obtained sleep have been indicated in previous ultra-marathon and solo ocean racing self-report studies [6,20,22]. Increases in the average sleep duration across the three distance categories suggest that sleep need increases as race duration (i.e., wake time) increases. This finding is consistent with the two-process model of sleep regulation [3]. That is, sleep is regulated by (1) a circadian process that increases the drive for sleep depending on time of day, and (2) a homeostatic process whereby the drive for sleep (i.e., sleep pressure) increases as sustained wakefulness increases [3]. It is clear that ultra-marathon competition impacts the homeostatic process of sleep regulation—Figure 2 depicts how both processes may be impacted during a 200-mile race.

Ultra-marathon athletes’ hesitance to sleep while racing is clear based on objective data [1], and in the subjective data from the present study. This is likely due to the dominance of either “do not sleep” or “sleep when exhausted” tactics demonstrated for in-race sleep strategies [1]. This “sleep when exhausted” mentality is likely impacted by the interplay between the circadian and homeostatic processes of sleep regulation [3]. The subjective feeling of exhaustion and/or extreme sleepiness is likely associated with periods of extended wakefulness coinciding with the lowest ebb of their circadian rhythm promoting sleep—i.e., when sleep pressure is highest (Figure 2). While it was not reported as an in-race strategy, it is reasonable to suggest that athletes could strategically reduce sleep pressure based on the two-process model of sleep regulation. This would require collection of pre-race habitual sleep data and/or circadian phase markers to estimate circadian rhythm [23,24,25], and ongoing measurement of prior wakefulness (i.e., time since last sleep opportunity).

Regarding post-race sleep strategy, it is encouraging to note that the majority of athletes value sleep as a post-race recovery tool. Almost 60% of athletes from the present study reported a strategy aimed at increasing sleep activity for a period following competition. The strategies specifically identified by athletes, such as night-time sleep extension and napping, are well-supported aids to recovery [7,16,19]. Of concern, some athletes reported no specific strategy regarding their habitual sleep behaviour following an ultra-marathon, especially for events longer than 100 miles. This post-race strategy may be problematic as athletes have already reported habitual nightly sleep below established recommendations of ~8 h [15]. As with some athletes in the pre-race phase of competition, a small percentage of athletes in the post-race phase may not be achieving adequate sleep outside of competition.

There are some boundary conditions that should be considered when interpreting the findings of this study. The subjective measures of sleep/wake behaviour utilised in this study are likely to be less accurate than the objective alternatives. However, it is difficult to obtain a larger sample size when utilising objective sleep/wake measurement during ultra-marathon events. Secondly, physiological measures of fitness were not acquired. It is possible that findings may differ between elite athletes and recreational athletes.

A factor that has not yet been investigated in ultra-marathon competition is the potential for circadian phase shifts during longer events. A phase shift is when the timing of the circadian component of sleep regulation moves earlier (phase advance) or later (phase delay) [23,26,27]. Light exposure prior to the low point of the circadian cycle results in a phase delay, while light exposure immediately following the low point of the circadian cycle results in a phase advance [28,29,30]. Recommendations for shifting phase using light exposure account for ~1 h shift per day [23,25]. Therefore, it is possible that ultra-marathon competitors could shift their circadian phase by approximately 3 h during races spanning over 3 ebbs of an athlete’s circadian rhythm. If future investigations are able to demonstrate shifts in the circadian phase during ultra-marathon competition, it is possible that these shifts can be used strategically to maximise performance. It should be noted that while based on established principles of chronobiology, it is not clear if the impact of phase shifters such as light would have the same impact during a high physical strain event. Future research should examine the impact of ultra-marathon competition on the circadian phase.

## 5. Conclusions

In addition to aligning with previous research concerning sleep strategies during ultra-marathon competition [6], the present study provides unique insight into strategies used prior to and after competition. For pre-race and post-race sleep, it is evident that athletes can improve their habitual sleep as well as implement strategies such as sleep extension or napping. Findings related to in-race sleep align with established sleep/wake behaviour models, suggesting that sleep need increases as wakefulness, or in this case, race duration, increases [3,4]. Athletes’ apparent aversion to in-race sleep may be at odds with their increasing sleep need during competition. This conflict indicates the importance of conducting field studies examining the effectiveness of in-race sleep management during ultra-marathon competition, particularly for races of longer duration. Future research should examine sleep timing, sleep duration, and the potential for changes in the circadian phase surrounding and during these events.

## Figures and Tables

**Table 1:** Self-Reported sleep data for as a function of race distance.

	Race Distance			Outcome
	**100–149 miles**	150–199 miles	≥200 miles	
**Variable**	(161–240 km)	(241–321 km)	(≥322 km)	
	n = 188	n = 28	n = 71	*p*
Race distance (km)	170.8 ± 16.8	252.8 ± 22.3	353.7 ± 44.4	<0.001
Race time (hours)	30.9 ± 8.3	41.0 ± 7.3	92.1 ± 23.8	<0.001
Sleep opportunities (count)	0.5 ± 2.1	2.5 ± 7.3	4.1 ± 3.2	<0.001
Sleep per opportunity (min)	10.6 ± 31.8	15.3 ± 31.9	59.3 ± 62.4	<0.001
Total sleep (min)	12.1 ± 50.5	45.4 ± 99.9	248.6 ± 228.0	<0.001

Notes: Post-hoc comparisons revealed significant differences between each race distance for all variables.

## Data Availability

Reasonable requests for the sharing of this data can be made directly to the authors.
